# Factors influencing practitioner adoption of carer-led assessment in palliative homecare: A qualitative study of the use of the Carer Support Needs Assessment Tool (CSNAT)

**DOI:** 10.1371/journal.pone.0179287

**Published:** 2017-06-16

**Authors:** Lynn Austin, Gail Ewing, Gunn Grande

**Affiliations:** 1 Centre for Primary Care, Faculty of Biological, Medical and Health Sciences, The University of Manchester, Manchester, United Kingdom; 2 Centre for Family Research, University of Cambridge, Cambridge, United Kingdom; 3 Division of Nursing, Midwifery and Social Work, Faculty of Biology, Medicine and Health, The University of Manchester, Manchester, United Kingdom; Public Library of Science, FRANCE

## Abstract

**Introduction:**

Informal caregivers play a pivotal role in supporting patients approaching the end of life. The Carer Support Needs Assessment Tool (CSNAT) is designed to facilitate person-centred assessment and support through a process that is practitioner-facilitated, but carer-led. This study explored practitioners’ experiences of implementing the CSNAT in palliative homecare.

**Methods:**

We conducted qualitative interviews/focus groups with 20 practitioners in one UK hospice homecare service (18 nurses, two healthcare assistants) before and after the implementation of the CSNAT. Thematic analysis of the data was underpinned by framework analysis.

**Results:**

Not all practitioners appreciated that using the CSNAT required a shift towards a more person-centred approach to assessment; consequently they tagged the tool onto their existing practitioner-led practice. Practitioners who did use the CSNAT as intended were able to act as role models and support their colleagues in making this transition. Practitioners’ comments revealed a number of contradictions: 1) Most felt that they ‘already do’ identify carer support needs, but feared using the CSNAT could increase their workload; 2) some worried about introducing the CSNAT ‘too soon’, but recognised that it was ‘too late’ once patients were close to the end of life; 3) whilst practitioners stated ‘they were there for the family as well as the patient’, care provision was overtly centred around patients.

**Conclusion:**

This study provides vital insights into barriers and facilitators to implementing the CSNAT as part of a person-centred approach to assessment. The findings identified the training and support required to help practitioners make this transition to this new way of working.

## Introduction

The pivotal role played by informal caregivers in supporting patients approaching the end of life (EOL) in a home care setting is recognised [1,2]. Following publication of the ‘Guidance on cancer services’ [3] the UK has seen a proliferation of policy and related publications highlighting the importance of supporting informal caregivers in general [4,5,6] and EOL in particular [7,8,9]. Collectively, these reports convey the need for a shift towards a more person-centred approach to assessment, comprising individual assessment of carers and a tailored response to needs identified by them.

Hospice services play a key role in carer support, given that they are there for the family as well as the patient [10]. However, whilst the importance of assessing and supporting individuals in their care-giving role is widely acknowledged, regular assessment of carers is yet to become embedded into routine hospice practice [11].

Whilst carer assessment tools exist, they often consider the impact of long-term caregiving [12]. Tools specifically for palliative care cover a range of carer experiences [13], however, they are often indirect measures of carer support needs, for example physical and mental wellbeing [14]. Direct measures, where they exist, tend to be lengthy [15,16,17] making them unsuitable for use in practice. In addition, none actively seek views of carers regarding which support needs are most important to them, and the type of support they would find helpful.

The Carer Support Needs Assessment Tool (CSNAT) was developed in response to the need for a comprehensive assessment tool that was suitable for use in practice by both carers and practitioners [18,19]. The tool comprises 14 broad domains which encompass areas in which carers commonly require support (Table 1). Carers are able to indicate the level of additional support they need (ranging from ‘no support’ to ‘very much more’) for any given domain. These domains reflect the dual role of carers as both providers of care for the patient and as clients in their own right [20,21].

**Table 1:** The CSNAT domains.

○ Understanding your relative’s illness ○ Having time for yourself in the day ○ Getting a break from caring overnight ○ Managing your relative’s symptoms, including giving medicines ○ Your financial, legal or work issues ○ Providing personal care for your relative (e.g. dressing, washing, toileting) ○ Dealing with your feelings and worries ○ Knowing who to contact if you are concerned about your relative (for a range of reasons, including at night) ○ Looking after your own health (physical problems) ○ Equipment to help care for your relative ○ Your beliefs or spiritual concerns ○ Talking with your relative about his or her illness ○ Practical help in the home ○ Knowing what to expect in the future when caring for your relative

The CSNAT provides a framework for a process of assessment which commences when the CSNAT is introduced to the carer. The carer is then given time to consider their support needs, using the domains as a prompt. A subsequent ‘assessment conversation’ with a practitioner enables the carer to prioritise their support needs and indicate the kind of support they would find helpful. This person-centred approach [22] facilitates joint action planning for key concerns in a tailored and timely manner. As such, the CSNAT is not simply a ‘form’ or a ‘tick-list’; it requires the practitioner to explore the carer’s own perspectives, rather than making assumptions about why certain domains have been highlighted.

The central tenet of the CSNAT is that it should be used as part of an assessment process which is facilitated by the practitioner, but led by the carer. Fundamental to this process is that the carers’ perspectives take centre stage not only in identifying support needs, but also suitable solutions. Consequently, incorporating the CSNAT into practice requires a fundamental, albeit subtle, shift into how carers’ support needs are identified and responded to.

We conducted a feasibility study to explore the potential role of the CSNAT in improving carer support in palliative home care. The aims were to:

1. explore practitioners’ experiences of implementing the CSNAT
2. use practitioners’ experiences to inform the development of a training guide to support implementation of the CSNAT

The study was successful in identifying the key barriers and facilitators to individuals’ adoption of the CSNAT and the implications for practice and training.

The views of family carers regarding the CSNAT were also sought; however these findings are beyond the focus of this paper.

## Methods

A qualitative study design was used. Data were collected via individual interviews and focus groups, and were supplemented by field notes.

### Sample/participants

The study was conducted in one UK hospice home-care service (Table 2).

**Table 2:** Characteristics of the study site/participants.

Two teams from the hospice participated: the Community Specialist Team (CST) and the Hospice at Home (H@H) team. These teams were based in a rural setting and collectively received around 100 referrals each month, the majority of whom were seen by the CST in the first instance. The average length of time patients spent on the caseload was 6 months, the H@H primarily providing respite care over the last few weeks of life. The CST comprised 18 Clinical Nurse Specialists (CNSs) plus one staff nurse; the H@H team had three nursing sisters and 15 HCAs. There was a single service manager for both teams. The 18 qualified nurses who participated in the study had been in post between 20 months and 12 years; 7 were educated to degree/diploma level. One HCA had been in post for 2 years and one for 8 years; both had received NVQ training. All participants were women

The CSNAT was implemented as part of service development, as such there was an organisational expectation that practitioners would incorporate the CSNAT into routine practice. Training was provided for all practitioners by the service manager and the authors. This one-hour session provided details about development of the CSNAT and guidance on how to use the tool in practice; it also provided practitioners with an opportunity to meet the researchers and gain an understanding of the purpose of the study.

The qualitative research ran in parallel to implementation. Whilst all practitioners were eligible to take part in the study, participation was voluntary. Invitations were sent to all 22 qualified nurses, of which18 participated (82%). Two HCAs who used the CSNAT also volunteered to take part.

### Data collection

Data were collected between February 2011 & January 2012. All authors were involved in data collection and field notes were made throughout the study (LA).

Focus groups of between 1–1¼ hours were held at three stages (Table 3) and guided by a topic list. Most people took part in one stage only for a variety of reasons, including clinical commitments, ill health, or leaving the organisation. However, two participants took part in all three stages; two participants in stages 1 and 2, and three participants in stages 2 and 3. One nurse was unable to take part in the round 2 focus group due to work commitments and participated in a short interview instead (12 minutes duration).

**Table 3:** Summary of focus groups/interviews.

Focus groups/interview	Dates held	Number of participants
1. Pre implementation focus groups (3)	February/March 2011	10
2. Post implementation focus groups/interview (3/1)	June 2011	8
3. Final focus group (1)	January 2012	11

Pre-implementation focus groups explored existing approaches to identifying carer needs, initial responses to the CSNAT and how staff anticipated using the tool in practice. The second round, four months post implementation focused on practitioners’ experiences of using the tool and the implications for training and support. The final meeting, 11 months post implementation acted as a closing session for the study. A number of recurrent themes emerged throughout this process with ‘data saturation’ of key themes.

### Data analysis

**Fig 1:**
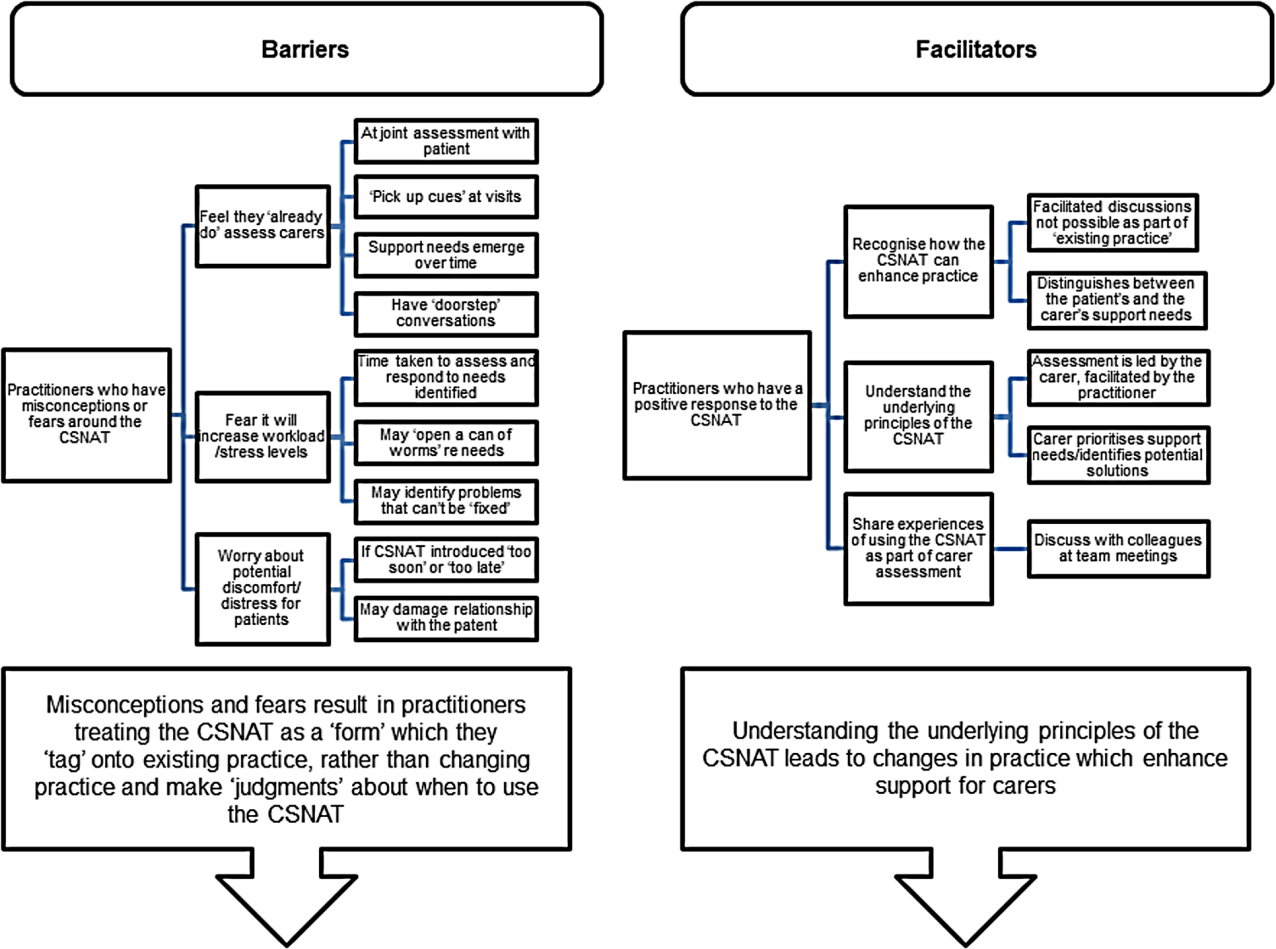
Barriers and facilitators.

Audio recordings were transcribed in full and thematic analysis conducted following the principles of framework analysis [23]. Transcriptions were read and re-read as part of the familiarisation process and reflective notes/memos made (LA & GE). Discussions amongst all authors facilitated development of a thematic framework for subsequent coding and charting of data (Fig 1). Preliminary findings were discussed with participants during the final focus group which provided the opportunity for reflection and clarification of emergent themes.

### Ethical considerations

University ethics approval was received from The University of Manchester Committee on the Ethics of Research on Human Beings on 6^th^ January 2011 (reference number 10308). Written consent was gained from participants when joining the study and reconfirmed at subsequent stages.

A unique alphanumeric code is used in the text to identify participants. We have adopted a generic term ‘P’ for all grades of staff to maintain participants’ anonymity.

### Rigour

The principles of rigour, pertaining to qualitative studies, were observed throughout the study; the COREQ criteria [24] have guided illustration of these principles within this paper.

## Results

The 20 participants comprised 14 CNSs, three sisters, one staff nurse and two HCAs who, collectively, had worked for the service between 20 months and 12 years. They provided information on existing practice relating to carer support, and barriers and facilitators to implementing the CSNAT. Their comments provided invaluable help for development of a training guide for CSNAT implementation.

### Existing practice

Practitioners stressed that they ‘already do’ identify carers’ support needs as part of usual practice. However, further discussion established that current approaches were informal and shaped by practitioners’ perceptions of carers’ needs, with ‘joint assessment’ of patients and carers being common:

**P20** We don’t actually use a tool when we do an initial assessment…although you have an agenda of your own…In your head are the things you need to extract from them to do your initial assessment.

Subsequent contacts with the family provided further opportunities for the carer’s needs to be raised. Practitioners noted the importance of developing relationships in order that carers would feel comfortable enough to discuss their concerns, allowing issues to emerge naturally:

**P24** I think as the patient’s journey goes on, and you’re building up a rapport with the patient and the carer, things crop up.

As part of this on-going relationship, exploration of concerns could be initiated by the practitioner who looked for ‘cues’ as to when the carer wished to talk, enquiries commonly framed in general terms such as ‘How are you?’ Carers could also initiate discussions, the frequently cited ‘doorstep conversations’ being the archetypal example:

**P10** Sometimes you’re stood on the doorstep. These are the ‘I don’t want them to know I’m talking to you’ conversations…that might be the time when you say to them ‘And how are you?’

### Barriers to implementation

#### Appreciation that a change to practice was required

Practitioners’ ‘initial response to the CSNAT was broadly favourable. The CSNAT domains reflected issues known to be of relevance to those in a caring role, as such, the CSNAT did not contain any ‘surprises’. However, comments indicated that the very resonance of the domains left some practitioners unclear as to how the tool could bring about tangible benefits:

**P21** In some ways I hope it doesn’t change our practice in that I hope we’re covering it all already, so if that is the case then it won’t hugely affect how we address things.

The comment above illustrates that some practitioners had not fully appreciated that implementation of the CSNAT required a shift from existing practice (which was essentially practitioner-led) to the approach required to incorporate the CSNAT (practitioner-facilitated, but carer-led). This perhaps accounts for P21’s description of how she attempted to ‘tag’ the CSNAT onto her existing practice:

**P21** I tend to do my whole assessment with the patient and the carer…as before, and give them information, at that point, as I would have before… but then say, “—and this is the form.”

The construction of the CSNAT as a ‘form’ or ‘paperwork’, as opposed to an opportunity for carers to express their support needs is of interest and has clear implications for training. This perception could inevitably shape how the CSNAT was introduced and subsequently interpreted by carers:

**P11** People don’t like forms, do they? They struggle with things, like the attendance allowance and if that was left to their own devices they wouldn’t fill that in half the time. So I think some people, they just get overwhelmed, don’t they, and it’s another form that somebody wants them to fill in.

Comments by others suggested that they construed the CSNAT as a ‘tick-list’ to demonstrate that topics had been discussed, e.g. during ‘joint assessments’. The primary benefit of the CSNAT being that, if nothing else, it made existing practice visible to others both within and beyond the organisation:

**P20** I think it does make additional work but, I think, in this day and age [with] …commissioning, and things like that, then, we have to be able to be seen to be providing that service and, perhaps, it helps us in doing that.

#### Concerns about changing practice

Tagging the CSNAT onto existing practice in order to document assessments was not uncommon during early stages of implementation. However, there was some recognition that the CSNAT required a shift in practice; most notably it required practitioners to make an overt distinction between the needs of the patient and the carer and ask about needs more directly. This led to some fears about the potential impact on workload due to the time required to undertake assessments and respond to support needs identified. One practitioner [P21] felt the overt assessment of carers could effectively double her caseload, others were concerned that extending the length of visits to accommodate separate assessment could be problematic for both the practitioner and the family:

**P10** I haven’t got negative feelings about [*the CSNAT*]. I worry a little bit that it might extend the period of time that we are there, which already can be quite a long time.

Furthermore, there was a fear that presenting carers with a predefined ‘list’ could trigger identification of previously unmet needs. Practitioners expressed discomfort with this as it could lead to carers having ‘unrealistic expectations’ as to what the hospice service could provide:

**P12** I was going to say it could impact on time if the carers are given that focus that we’re there for them and…they may realise that they do need more and so we may actually end up giving more of our time to the carers, as well as the patients. So that could impact on the workload.

The fear of ‘opening up a can worms’, in relation to needs identified, was related to discomfort felt by practitioners when presented with a concern that they may not be able to respond to or ‘fix’, the following practitioner giving a very open account of how she might feel:

**P10** I don’t know if it’s a nurse thing or what, but…if I’m going to be picking up things on here that I can’t fix that might make me feel uncomfortable…I know we can’t fix everything and I’m used to that day in/day out, but [*the CSNAT*] will make it very much in my face.

Concerns were also expressed about appropriate timing of assessment both pragmatically and with regard to the impact on the carer and the practitioner-carer relationship. The first visit was commonly cited as ‘too soon’ as the patient would inevitably be the focus of attention for both practitioner and carer:

**P14** You would be more focused on the patient, possibly. Not saying that the carer isn’t important, but…getting the patient details is obviously more pertinent…To me that would be more a priority than filling out even more paperwork.

Conversely, introducing the CSNAT to carers of patients close to the end of life was frequently viewed as inappropriate, given the need to assess and respond to the patient’s needs in the short space of time available:

**P11** I’ve felt more comfortable leaving [*the CSNAT]* at earlier stages…I’ve struggled, perhaps, more when I’ve gone in to meet someone who is, actually, very much approaching end-of-life…I’ve always felt leaving the form gives [*the carer*] something else to worry about.

Finding a suitable point to introduce the CSNAT was dependent, nominally, on how long the family was likely to be in contact with the service and frequency of visits. However, some practitioners were concerned that, if introduced at the ‘wrong’ time, the CSNAT could distress carers by ‘forcing’ them to think about issues before they were ready, or even cause them to worry about things that they hadn’t anticipated:

**P14** It might bring things up as well that maybe at that particular time the carer’s not prepared to, or doesn’t want to, identify…So, I don’t know, by asking that are you bringing things out that they are not ready to discuss?

This ‘gatekeeping’ role with regard to when to introduce the CSNAT was driven by both practicalities, such as visiting patterns, and a desire to be sensitive to the perceived readiness of the carer to discuss some issues. It was feared that introducing the CSNAT at the wrong time would not only fail to elicit carers’ support needs, it could also distress the carer and potentially damage a fledgling relationship:

**P11** You’ve almost got to try and maintain that relationship…and keep that foot in the door to try and be there throughout and I think, sometimes, if you try and push too much or encourage too much, it can be to the detriment of that relationship so…sometimes, then, filling forms in doesn’t feel appropriate…I think it’s that gut instinct…and our experience that actually helps you make that decision as to whether it’s actually going to be helpful or not.

### Facilitators to implementation

As seen, barriers to implementation related, largely, to the extent to which practitioners felt the CSNAT could enhance existing practice. Consequently, practitioners frequently used their judgement as to whether the tool would be beneficial in a particular circumstance, as opposed to using routinely for all carers. However, a number of practitioners did perceive the CSNAT as an enhancement to existing practice and were able to incorporate the tool in a meaningful way. The following practitioner gave a clear example of how using the CSNAT overcame the limitations of existing practice:

**P21** on one occasion, a patient said to me, oh, she wants to go through the [*CSNAT*] with you…the relative had never previously made any move to talk to me, or sit with me, or be part of the conversation. So there are conversations in which it has been really helpful in just giving that opportunity, which I might not otherwise have had’

Similarly, some respondents felt the CSNAT had improved their existing practice by acting as a tool to facilitate communication:

**P14** Now that we are going through this…the carers are having the questions put to them rather than the CNS picking it up in conversations, more of a direct [*other agreeing*] you know… ‘do you need more support with -?’ It’s more of a direct contact than just picking it up in little snippets.

Those who felt positive about the CSNAT appeared to have understood the underlying principles of the approach which distinguished carers’ and patients’ needs and allowed the carer to prioritise their support needs and identify potential solutions:

**P03** I think it's good in a sense that it gets them, not us—because we can go in and say you need this, this and this, but this isn't designed for the patient's needs, it's designed for the carer's needs. So I think it's good that it gets them to prioritise what they feel their needs are, certainly not what we think.

These natural role models were able to champion this person-centred approach to assessment. For example, the focus groups provided an opportunity for these natural ‘champions’ to share examples of how they had integrated to CSNAT into their way of working and enhance carer support. These champions also used the focus groups to help problem-solve issues or challenge perceptions. This can be seen in the following exchange where practitioner P11 responds to a colleague’s concerns:

**P20** When they’re looking at the [*CSNAT*] questions that say “Do you need more advice on help with your personal care?” and things like that, they don’t want to look at what might be happening in the future…it’s the here and now. And that actually sometimes makes them think about; “well, does this mean that…I’m not going to be able to stay on my own at night?” and things like that.

**P11** But I guess turning it around, it may well be reassuring for some families to actually talk about; “well actually, if I got to that point and I did need help, [I’d] know who to ring, or what support might be available.”

These informal exchanges took place on other occasions, including where practitioners shared office space or attended team meetings. These conversations enabled colleagues to reflect on how they were using the CSNAT, share examples of good practice and support each other during the transition to this new way of working.

## Discussion

Participants provided invaluable insights into their existing practice regarding identification of carers’ support needs, their perceptions of the CSNAT, and their feelings about using the tool. Collectively, the factors which facilitated or acted as barriers to implementation fed directly into the training developments.

Many practitioners recounted occasions on which the tool proved beneficial [25]. However, the extent to which the CSNAT was utilised was dependent on a range of factors which could impact on meaningful integration into practice. A key factor seemed to be whether individuals understood the underlying principles of the CSNAT and could see how it could enhance existing practice.

Use of the CSNAT was also affected by practical issues such as workload, visiting patterns, sensitivities around potential discomfort for carers, and the extent to which carers’ needs could be accommodated separately from those of the patient. Consequently, for a number of practitioners, practice in relation to carer assessment remained largely unchanged; the CSNAT simply acting as a ‘form’ for recording ‘existing practice’.

Understandably, it could take practitioners time to become fully acquainted with how to use the tool. However, some practitioners appreciated the potential benefits of the CSNAT at an early stage. These role models were willing to engage with their colleagues in group settings to help them overcome perceived problems in relation to the implementation of the CSNAT.

### Contradictions

Interestingly, discussions with practitioners revealed what appeared to be a number of contradictory comments; these related to identifying carers’ needs, when to introduce the CSNAT, and the carer as a ‘client’ of the service. The following sections explore three of these apparent contradictions alongside the literature which helps to illuminate them.

#### Contradiction one: “We already do” identify carers support needs versus fear of increasing workload

The CSNAT did not, of course, arrive in a vacuum, however, existing practice was informal and practitioner-led, reflecting practice in hospice services more widely [26]. Given that practitioners said they routinely take account of carers’ support needs, the potential for an increase in workload, should the CSNAT be adopted, was an unexpected finding. This fear seemed to emanate from concern that, in separating out the carers’ needs from those of the patient and providing carers with the overt opportunity to express their support needs, more issues would need to be resolved.

The concerns suggest that there were some misconceptions about the tool. For example, the perception that the CSNAT will ‘open up a can of worms’ and identify issues that the practitioner would then need to ‘fix’ runs counter to the idea of a carer-led, practitioner-facilitated approach in which the carer prioritises their needs and helps to identify potential solutions.

The desire for practitioners to ‘fix’ problems and feelings of guilt if they are unable to is a recognised phenomenon [27,28]. Whilst understandable in a caring profession, this can have the negative effect of limiting what is discussed for fear of causing distress. Consequently, practitioners require reassurance that simply acknowledging and discussing a carer’s concerns, even if not readily resolved, may be helpful in its own right. Likewise allowing carers to identify their support needs would not necessarily lead to limitless demands [15].

#### Contradiction two: It’s ‘too’ soon to introduce the CSNAT versus it’s ‘too late’

For some, the fear that the CSNAT could increase workload was enough to limit use of the tool. However, even practitioners who planned to introduce the CSNAT found it difficult to find a suitable ‘window of opportunity’. Whilst practical issues such as visiting patterns came into play, the overriding factor seemed to be sensitivities around when it was appropriate to broach potentially distressing topics. There was a misconception that the CSNAT might force people to consider issues before they were ready, as opposed to the domains legitimising concerns and providing the opportunity for carers to open up a conversation about topics important to them. Conversely, if the patient was very close to the end of life it was felt to be ‘too late’ to discuss the carer’s needs given the speed at which events were occurring.

Determining the appropriate time to open up ‘difficult’ conversations is not unique to carer assessment. Boot and Wilson [29] found that introducing the topic of Advance Care Planning was influenced by practitioners’ assessment of the patient’s readiness to discuss the topic’ and whether a relationship had been established with the patient. Fields et al [30] also found that practitioners spoke about their own emotions as to why they avoid difficult conversations around someone’s preferred place of death. Practitioners looked for cues as to when patients might wish to discuss this as, if raised too soon, any distress caused might damage the relationship. Fields et al [30] contest this view, suggesting that difficult conversations can bring people closer and that people prefer clinicians who are willing to initiate these types of discussion.

Developing relationships was valued as practitioners believed that carers’ needs would come to the fore as the relationship progressed. Hill et al [31] challenge the view that ‘familiarity is either a necessary or sufficient condition for the provision of psychosocial support’. Their study of registered and auxiliary nurses in a palliative care setting found that familiarity may result in ‘unwarranted assumptions’ which block rather than facilitate support. Hill et al [31] suggest that it’s the formation of an interpersonal connection with the client that is important, and this can occur even on initial contact. This is viewed as particularly important in view of the relatively short time frame that may be available for these exchanges, given the palliative care context. This view is echoed by Payne and Morbey [11] who note that ‘the window of opportunity for carers to access appropriate information and support is often brief.’

#### Contradiction three: ‘We’re there for the family as well as the patient’ versus the patient is the ‘priority’

Whilst services were nominally ‘there for the family as well as the patient’, it was clear that this was subject to a number of provisos. Although ‘joint assessments’ took place, patient’s needs were clearly prioritised. Likewise, patient assessment formed part of routine practice, whereas carer ‘assessment’ was less overt and subject to the practitioners’ perception of whether it was appropriate. Explicit provision for carers was largely viewed as ‘extra’, as revealed by the comment that the caseload would ‘double’ if carers’ needs were to be assessed separately from those of the patient. This leads to the question as to whether existing practice constitutes ‘assessment’.

### Implications for person-centred assessment and practice

Whilst carer assessment has been much discussed in policy and professional literature, guidance on how this can be achieved is relatively sparse [26]. In contrast, patient assessment has received much attention and has long been accepted as a core component of practitioners’ work. Recent palliative care publications highlight good practice in relation to holistic nursing assessment [7,32]. These contemporary views favour assessment in which patients identify their support needs and work towards joint solutions with practitioners in order for responses to be tailored to the patient’s individual needs. As such these patient-centred approaches to assessment have much in common with the underlying principles of the CSNAT, and indeed issues we encountered as part of this study have resonance with those highlighted in relation to patient-centred assessment [7].

The apparent contradictions raised in this discussion can be understood when viewed in the light of how ‘existing’ practice enables practitioners to contain some of the demands placed on them. Whilst practitioners ‘already do’ consider carers’ needs, the extent to which these are explored is limited by time constraints, fear of causing distress, and the conflation of patients’ and carers’ needs. That is, practitioners exert some control over what is discussed, who it is discussed with and when. Consequently, practitioners’ sensitivity to carers’ support needs did not necessarily translate into an opportunity for carers to express these needs in full. In view of these findings it is perhaps not surprising that practitioners may have had reservations about incorporating the CSNAT into routine practice. When used as intended, the CSNAT clearly shifts the locus of control to the carer, allowing them to shape the assessment process and determine which support needs they would like to discuss and when.

In summary, adoption of the CSNAT was not straightforward for many practitioners as they had an established working pattern which helped them manage demands placed on them whilst being sensitive to the carers’ needs. Implementing change is not straightforward, a prerequisite to success being that the change desired is viewed as beneficial [33].The CSNAT faced the dual challenge of not always being perceived as overtly helpful and also as having potential problems. These concerns were based primarily on misconceptions of the CSNAT’s underlying principles.

The misconceptions of some practitioners have implications for practice as it is clear that practitioners require time to make the transition to this new way of working. Consequently, the means by which the underlying tenets of the CSNAT are conveyed to practitioners requires further consideration if successful implementation of the tool is to enhance practice. As a result of this study the training and on-going facilitation relating to implementation have been substantially revised (Table 4). In particular the ‘person-centred’ approach underpinning the process of assessment is highlighted. In addition, the ‘external’ facilitation required from the ‘CSNAT team,’ and the ‘internal’ facilitation required from the organisation, to support this transition to a new way of working, are more clearly structured.

**Table 4:** Lessons learnt and implemented as a result of the feasibility study.

**External facilitation from the CSNAT team**
a) Initial training
Following the initial feasibility study the CSNAT was rolled out to six hospices as part of a further study. The original training sessions were extended and revised in order that more information could be provided on the evidence-base of the tool and how it could be used to enhance existing practice. Emphasis was placed on the difference between existing informal, practitioner-led, approaches to carer assessment and the CSNAT, which provides structure for a carer-led, practitioner-facilitated approach. The primary message conveyed was that whilst the CSNAT provides a framework for the assessment process, it is not simply a form. Vignettes were also shared, which give ‘real life’ examples of how of how the CSNAT has achieved successes that would not have been possible with ‘existing practice’.
b) On-going facilitation
Following the initial training sessions a further follow up visit was made to each participating site around six weeks post implementation. This meeting with practitioners provided the opportunity for them to discuss how things were progressing in relation to the implementation of the CSNAT, this allowed for any queries or misconceptions to be discussed at an early stage. Provision was made for a CSNAT ‘champion’ who acted as an internal facilitator for the CSNAT within their service and acted as a link between the organisation and the CSNAT team. Support for the champions was facilitated by the CSNAT team via a series of one-to-one phone calls with the individual champions and provision of occasional ‘Skype’ and face to face meetings with fellow champions. **Internal facilitation from the organisation**
a) Organisational preparation
The lessons learnt from the study were incorporated into an advisory document which identified the organisational preparations required prior to implementation of the CSNAT, in particular the internal facilitation processes that needed to be in place. Attention was drawn to the need for a ‘change management’ approach to be considered to support implementation.
b) Core group
Sites were advised to have a ‘core group’ of individuals who could help steer the implementation. This core group comprised the champion, service manager and an administrator. Sites were also encouraged to frame the implementation as a service development by which new evidence on carer assessment could enhance practice and build on existing skills. Sharing of experiences of using the CSNAT was encouraged in order that practitioners could support each other and ‘early adopters’ of this new approach could illustrate how this tool enhances existing practice and any potential hurdles to implementation could be overcome.

### Limitations

The study provides key information relating to the adoption of the CSNAT by individual practitioners working within hospice homecare. Whilst some information was gained on the contextual factors practitioners work within, this was not extensive. Context can clearly play a key role in the implementation of a new way of working. Hospices show wide variation in both the size and nature of services they provide and the composition of teams. Further work has now been undertaken with a larger group of services which will provide more detailed information on key contextual factors which impact on the successful integration of the CSNAT.

## Conclusion

The study highlights a number of factors influencing uptake of the CSNAT into routine practice. The findings revealed that, as with any tool, the CSNAT it is not a ‘quick fix’ solution to assessing carers’ needs. In order for the tool to be meaningful it needs to be used as part of a person-centred approach to assessment. Practitioners need training and support to make a transition from existing practitioner-led practice to one which incorporates a carer-led approach. Whilst this new approach builds on existing skills it differs from what practitioners ‘already do’. Transition to this new way of working needs to be underpinned by training in the underlying principles of this approach and requires on-going facilitation until this new way of working is embedded into routine practice.
